# Identification of Superior Haplotypes and Haplotype Combinations for Grain Size- and Weight-Related Genes for Breeding Applications in Rice (*Oryza sativa* L.)

**DOI:** 10.3390/genes14122201

**Published:** 2023-12-12

**Authors:** Gang Liu, Dongfeng Qiu, Yuxia Lu, Yan Wu, Xuesong Han, Yaru Jiao, Tingbao Wang, Jinsong Yang, Aiqing You, Jianguo Chen, Zaijun Zhang

**Affiliations:** 1 Key Laboratory of Crop Molecular Breeding, Ministry of Agriculture and Rural Affairs/Hubei Key Laboratory of Food Crop Germplasm and Genetic Improvement, Institute of Food Crops, Hubei Academy of Agricultural Sciences, Wuhan 430064, China; liug1112@hbaas.com (G.L.); 18672779158@hbaas.com (D.Q.); luyuxia0812@163.com (Y.L.); yanwu@hbaas.com (Y.W.); hanxuesong@hbaas.com (X.H.); jyr20230130@163.com (Y.J.); tb_wang7777@163.com (T.W.); yang_js@hbaas.com (J.Y.); aq_you@hbaas.com (A.Y.); 2 Hubei Hongshan Laboratory, Wuhan 430070, China; 3 School of Life Sciences, Hubei University, Wuhan 430062, China

**Keywords:** rice, candidate gene-based association analysis, grain size, thousand-grain weight, superior haplotype

## Abstract

The identification of superior haplotypes and haplotype combinations is essential for haplotype-based breeding (HBB), which provides selection targets for genomics-assisted breeding. In this study, genotypes of 42 functional genes in rice were analyzed by targeted capture sequencing in a panel of 180 *Indica* rice accessions. In total, 69 SNPs/Indels in seven genes were detected to be associated with grain length (GL), grain width (GW), ratio of grain length–width (L/W) and thousand-grain weight (TGW) using candidate gene-based association analysis, including *BG1* and *GS3* for GL, *GW5* for GW, *BG1* and *GW5* for L/W, and *AET1*, *SNAC1*, *qTGW3*, *DHD1* and *GW5* for TGW. Furthermore, two haplotypes were identified for each of the seven genes according to these associated SNPs/Indels, and the amount of genetic variation explained by different haplotypes ranged from 3.24% to 27.66%. Additionally, three, three and eight haplotype combinations for GL, L/W and TGW explained 25.38%, 5.5% and 22.49% of the total genetic variation for each trait, respectively. Further analysis showed that Minghui63 had the superior haplotype combination Haplotype Combination 4 (HC4) for TGW. The most interesting finding was that some widely used restorer lines derived from Minghui63 also have the superior haplotype combination HC4, and our breeding varieties and lines using the haplotype-specific marker panel also confirmed that the TGW of the lines was much higher than that of their sister lines without HC4, suggesting that TGW-HC4 is the superior haplotype combination for TGW and can be utilized in rice breeding.

## 1. Introduction

As the most important crop worldwide, rice (*Oryza sativa* L.) plays a key role not only in agriculture but also in the economy. The grain yield of rice is a complex trait that is determined by the number of panicles, the number of grains per panicle and grain weight [1]. Among these factors, grain weight is determined by grain size directly, as specified by GL, GW and L/W, and is also a major determinant of rice quality. Therefore, grain size is always an important selection target during rice domestication and breeding [2,3]. Breeders tend to cultivate rice varieties with appropriate grain sizes for milling quality and market preferences.

The identification of genes conferring grain size variation will provide valuable targets in molecular breeding procedures. During the past few decades, tremendous progress has been made in the functional genomics of rice, and a number of grain trait-related QTLs/genes have been cloned. For instance, *GS3* is a major QTL for grain length and weight, which encodes a putative transmembrane protein and functions as a negative regulator of grain size [4,5]. *GW5* functions in the ubiquitin–proteasome pathway to regulate cell division during seed development and grain width formation [6,7]. *GIF1* encodes a cell wall invertase, which can improve grain weight by increasing grain filling [8]. *GS5* encodes a putative serine carboxypeptidase and functions as a positive regulator of grain size by regulating grain width, filling and weight [9]. Higher expression of *OsSPL16*, which encodes a protein that is a positive regulator of cell proliferation, promotes cell division and grain filling, with positive consequences for grain width and yield in rice [10]. *BG1* encodes a novel membrane-localized protein that suggests its role in regulating auxin transport and can increase plant biomass, seed weight and yield [11]. The functional gene for *qTGW3* is *OsSK41*, which is a member of the GLYCOGEN SYNTHASE KINASE 3/SHAGGY-like family and controls the grain size and weight of rice [12,13,14]. LG1 encodes a constitutively expressed ubiquitin-specific protease 15 (*OsUBP15*), which is also a positive regulator of grain width and size in rice [15]. These functional genes for grain size will be of great importance for realizing high yield potential.

With the progress in rice functional genomics and high-throughput sequencing technologies, rice breeding has evolved from conventional to genomics-assisted breeding (GAB). GAB approaches are strategies that use genomics tools and technologies to identify markers and candidate genes that are associated with target traits and then integrate them into breeding [16]. Several GAB approaches, including haplotypes-GWAS, genomic selection (GS) and haplotype-based breeding (HBB), which pyramid favorable alleles or haplotypes together, have been used in crop improvement [17,18]. HBB is a new approach for crop improvement, which, together with the phenotyping data of germplasm/breeds, is used to assess and validate the phenotypic effects of the ‘component’ haplotypes [19]. Based on the haplo-pheno analysis approach, useful haplotypes are identified for crop breeding. For example, Sinha et al. [20] identified superior haplotypes for 10 drought-responsive candidate genes in pigeonpea. Based on genome-wide association studies, 11 genes were selected for haplo-pheno analysis to identify the superior haplotypes for resistant starch, the glycemic index and grain and cooking quality in rice [21]. Liu et al. [22] evaluated the effects of 65 genes related to rice zinc responses on grain zinc content using two panels of breeding lines, and superior haplotypes were identified. Abbai et al. [23] conducted a candidate gene-based association study for 120 genes and identified 21 strongly associated genes governing 10-grain yield and quality traits and identified superior haplotypes for the associated genes upon phenotyping the subset of the 3k rice genome panel.

In the present study, genotypic variations of 42 genes in 180 *Indica* rice accessions were examined by targeted capture sequencing, and SNPs/Indels associated with grain features were identified using candidate gene-based association analysis. Additionally, superior haplotypes and superior haplotype combinations for the associated genes were also identified, which will be useful for breeding rice varieties with high yield potential and good quality by the HBB strategy in the future.

## 2. Materials and Methods

### 2.1. Plant Materials

A panel of 180 *Indica* rice accessions with great yield variation was provided by the National Key Research and Development Program of China ‘Precise identification and innovative utilization of *Indica* rice germplasm resources in Central China (2016YFD0100101-05)’, including 89 modern varieties, 39 advanced generation lines, 18 landraces, 18 restorer lines and 16 foreign germplasms from other countries (Table S1). All of these accessions are preserved in the National Crop Genebank of China, which is under the charge of the Institute of Crop Sciences, Chinese Academy of Agricultural Sciences.

### 2.2. Field Experiment and Agronomic Trait Investigation

The field experiments were conducted at the experimental station of Hubei Jingzhou Academy of Agricultural Sciences (30°23′ N, 112°35′ E) in 2018 and 2019. All of the accessions were sown on May 16th. In total, 100 plants for each accession were transplanted into a plot one month later with a spacing of 25 cm between rows and 15 cm between plants, and it was maintained within a 1.5 m long row. The management of the field experiments was performed in accordance with local standard practices. One plant from the middle and the four corners of the plot (totaling five plants) for each accession was harvested at maturity and dried under natural conditions. Grains of the five plants were pooled together. Grain length (GL), grain width (GW), the ratio of grain length–width (L/W) and thousand-grain weight (TGW) were investigated using the rice Digital Yield Traits Recorder (YTS-RICE-04D) provided by Wuhan Hongxingyang Technology Co., Ltd. Heritability was calculated using QGA Station software (V. 2.0) (http://ibi.zju.edu.cn/software/qga/ (accessed on 25 Februry 2023)) by the formula *h*^2^ = *Vg*/(*Vg* + (*Vge*)/*e* + *Ve*/*re*), in which the respective variance components are attributed to genotypic, genotype environment and experimental error effects, *r* is the number of replicates per environment, and *e* is the number of environments for a given trait. Correlation analyses and data visualization for these traits were conducted by *R* (v. 4.0.5) package “corrlpot”.

### 2.3. DNA Extraction and Library Construction

The genomic DNA of the 180 rice accessions was extracted using a plant DNA extraction kit according to the manufacturer’s instructions (Tiangen, Beijing, China). The DNA concentration was determined using a Nanodrop spectrophotometer (Thermo Scientific, Waltham, MA, USA) according to the manufacturer’s instructions. DNA quality and purity were further checked on a 0.8% agarose gel.

Two hundred nanograms of genomic DNA from each accession were physically sheared to an average size of 150–200 bp by a Biorupter (Diagenode, Belgium). The ends of the DNA fragments were repaired, and an Illumina adaptor was added to generate dual-indexed libraries (Fast Library Prep Kit, iGeneTech, Beijing, China).

### 2.4. Probe Design and Targeted Enrichment Assay

A custom target enrichment sequencing assay including 42 target genes that have great potential utilization value in rice breeding was performed, including 15 genes for grain size, 5 for quality, 11 for heading date, and 11 for environmental stress resistance (Table S2). The coding genomic region and upstream region (−2000 bp) of the 42 genes (totaling 268,132 bps according to reference sequences of the 42 genes on the genome of Nipponbar) were extracted from the Rice Genome Annotation Project (http://rice.plantbiology.msu.edu/ (accessed on 25 March 2022)) using the gene ID as the query. Customized overlapped probes were then designed based on the whole gene sequence to capture all the variations in the gene coding region and regulatory region. Hybridization of customized DNA baits with capture pools was performed at 65 °C for 24 h according to the manufacturer’s instruction (Beijing Igenecode Technology Co., Ltd., Beijing, China). Then, the target regions were hybridized with customized probes for each gene. Based on these probes, captured libraries were obtained, mixed in equal molar amounts, and sequenced with the sequencing depth being 500× on the Illumina platform (Illumina, San Diego, CA, USA) with 150 bp paired-end reads.

### 2.5. Reference Genome Alignment and Variation Analysis

All resulting clean reads were aligned to the reference genome (Version IRGSP-1.0) using BWA (Burrows Wheeler Aligner) software [24]. Then, the Picard tools tag was used to remove duplicate reads. The HaplotypeCaller tool of GATK (v. 4.0.3.0) [25] was used to detect SNPs and Indels, and the method of hard filtering (QUAL < 30, QD < 4.0, FS > 60.0, MQ < 40.0) was used to obtain high-quality and reliable variant sets for downstream analysis. The snpEff (v. 4.3) [26] program was used to identify the locations of these SNPs and Indels, including intergenic regions, gene regions or CD regions, and to clarify whether they were frameshift variations.

### 2.6. Phylogenetic and Population Genetics Analyses

Using PLINK (parameters: --indep-pairwise 100 50 0.2), pruning was then conducted to reduce variant redundancy caused by linkage disequilibrium (LD), and the non-redundant variants were subjected to further analysis. An unrooted neighbor-joining phylogenetic tree was then built using MEGA-X [27] with 1000 bootstraps. Principal component analysis (PCA) was performed with the final variant dataset using GCTA (genome-wide complex trait analysis, v. 1.26.0) [28]. Population structure analysis was carried out using STRUCTURE (v. 2.3.4) [29]. STRUCTURE analyses were run 10 times for each *K* value from 1 to 9 using the 394 extracted variants. The length of the burn-in period and the number of MCMC repetitions after burn-in were both set to 10,000. The STRUCTURE outputs were summarized using STRUCTURE HARVESTER to identify the most likely value of *K* [30]. After the optimal *K* was determined, the population structure of the accessions was inferred using fastStructure (version 1.0) [31] with the final variant dataset for each *K*.

### 2.7. Candidate Gene-Based Association Analysis

The final variant dataset of the entire population was used for candidate gene-based association analysis. Kinship matrices of relatedness between the accessions were calculated using the “-gk” function of Genome-wide Efficient Mixed-Model Association (GEMMA) with the default option. The matrices were then used to correct the population structure. The association analysis was performed using GEMMA, which was designed to handle large dataset analysis [32]. Marker and trait associations with *p* values < 0.01 were considered significant.

### 2.8. Identification of Superior Haplotypes and Haplotype Combinations for the Associated Genes

To identify superior haplotypes for the associated genes, the accessions were divided into several groups according to their haplotypes or haplotype combinations for the associated genes, and the groups with more than three accessions were further analyzed. Student’s *t*-test and Duncan’s test were used to analyze the difference between the phenotypic mean values of the different haplotype or haplotype combination (HC) groups, while the boxplots were drawn with *R* (v. 4.0.5) package ”ggplot2”. Superior haplotypes for the associated genes were then evaluated according to their contribution to the target traits. The penta-primer amplification refractory mutation system (PARMS) [33] was used to develop a haplotype-specific marker panel for the haplotype combinations of TGW.

## 3. Results

### 3.1. Phenotypic Variation Analysis

Four grain size-related traits, namely, GL, GW, L/W and TGW, were investigated and statistically analyzed for the 180 *Indica* rice accessions in 2018 and 2019. The BLUP value of the four traits based on the two years of data revealed high heritability for all of them, which was 0.94 for GL, 0.86 for GW, 0.91 for L/W and 0.80 for TGW (Table S3).

**Figure 1:**
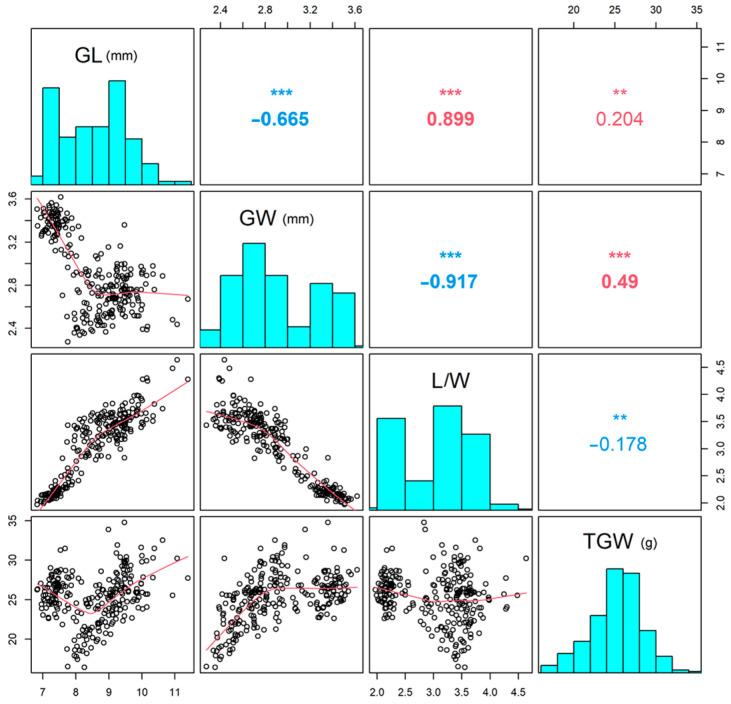
Correlation analysis of 4 target traits in the 180-rice accession panel. The lower triangle in the figure represents the scatter plot of the target trait, the diagonal line represents the histogram of the target traits (the scale indicates phenotype value of the traits), and the upper triangle represents the relationship between the traits. Correlation coefficient, ** *p* < 0.01, *** *p* < 0.001.

Correlation analysis of BLUP values between the four traits (Figure 1) showed that GL was negatively correlated with GW (*p* < 0.001) but positively correlated with L/W (*p* < 0.001) and TGW (*p* < 0.01). Furthermore, a significant negative correlation was detected between GW and L/W (*p* < 0.001), but a significant positive correlation was observed between GW and TGW (*p* < 0.001). Although both GL and GW were positively correlated with TGW, GW contributed to TGW more than GL, and the correlation coefficient between GW and TGW was 0.49, while it was 0.204 between GL and TGW. Moreover, L/W showed a significant negative correlation with TGW (*p* < 0.01).

### 3.2. Targeted Enrichment Analysis and Sequence Variation of the 42 Genes in the Population

The capture assay was designed to capture a total of 268,132 bp of the 42 target genes (including the coding regions and 2000 bp upstream regions) in the 180 rice accessions. A total of 98.82% of these clean reads could be anchored to the rice genome, and a total of 1,718,956 effective bases (approximately 202.29 Mb) were obtained after excluding the duplicated reads. Approximately 64.01% (capture specificity) of these effective bases could be anchored to the target regions. The average depth of the target regions sequenced was 482.73×. An average of 99.42% of the target regions was covered by at least one read in each accession, and 98.63% was covered by at least ten reads. Finally, a total of 2742 variants (2164 SNPs and 578 Indels) with high quality for the 42 genes within the 180 rice accessions were identified using the BWA and GATK software, which were subjected to further analysis.

### 3.3. Population Structure

**Figure 2:**
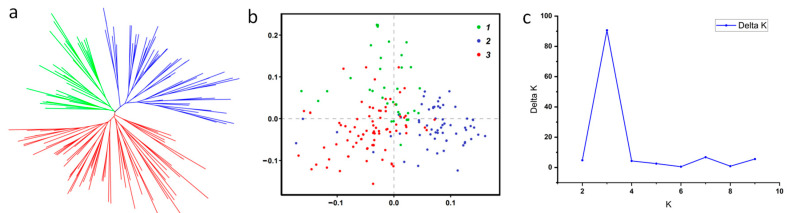
Population analysis of the 180 accessions. (**a**) SNP and Indels-based unrooted neighbor-joining phylogenetic tree reveal the presence of three major clusters, (**b**) two significant principal components were found in the panel; 1, 2 and 3 represent subpopulations 1, 2 and 3, respectively, (**c**) estimated Delta K from structure analysis.

To further understand the phylogenetic relationship and population structure of the 180 rice germplasms at the genomic level, phylogenetic analyses (Figure 2a), principal component analyses (PCAs, Figure 2b) and population structure analyses (Figure 2c) were performed based on their genotype information. Further analysis showed that the 180 germplasms could be divided into three distinct subpopulations according to their genotypic data for the 42 target genes, which included 39 (subpopulation 1), 58 (subpopulation 2) and 83 (subpopulation 3) accessions, respectively. Moreover, it seems that accessions from the same region of China are not clustered in the same subgroup (Table S1).

### 3.4. Association Analysis

Association analysis between the 2164 SNPs and 578 Indels in the 42 genes and grain traits was then conducted based on the grain trait performance of the 180 accessions in 2018 and 2019, as well as their BLUP values. SNPs/Indels could be detected in both years and by BLUP value, they were considered to be the loci associated with the target traits. Consequently, 56 SNPs and 13 Indels in seven genes were identified to be significantly associated with the four grain traits, including *BG1* and *GS3* for GL, *GW5* for GW, *BG1* and *GW5* for L/W, and *SNAC1*, *qTGW3*, *GW5*, *AET1* and *DHD1* for TGW. It was notable that the same SNPs in *BG1* were associated with both GL and L/W, and the same SNPs in *GW5* were associated with both GW and TGW.

We also found that the location of these associated SNPs in the gene region or in the upstream or downstream region varied greatly for different genes. For instance, the four SNPs in *BG1* were located in the 5′UTR or promoter region, 31 SNPs in *GS3* were located in the 3′UTR, exons, introns, the 5′UTR or promoter regions, 7 SNPs for *GW5* were located in the promoter or exons, the only SNP detected in *SNAC1* and *AET1* was located in the promoter region, and the only SNP in *DHD1* was located in the exon. It was noteworthy that at least one associated SNP was located in the promoter or exon region of all the six other genes except for *qTGW3*, in which all the associated SNPs were located in the intron or 3′UTR. It was interesting that all of the SNPs in the exons of the target genes resulted in nonsynonymous mutations or premature stop codons (Table S4).

### 3.5. Haplotype Analysis and Identification of Superior Haplotypes

**Figure 3:**
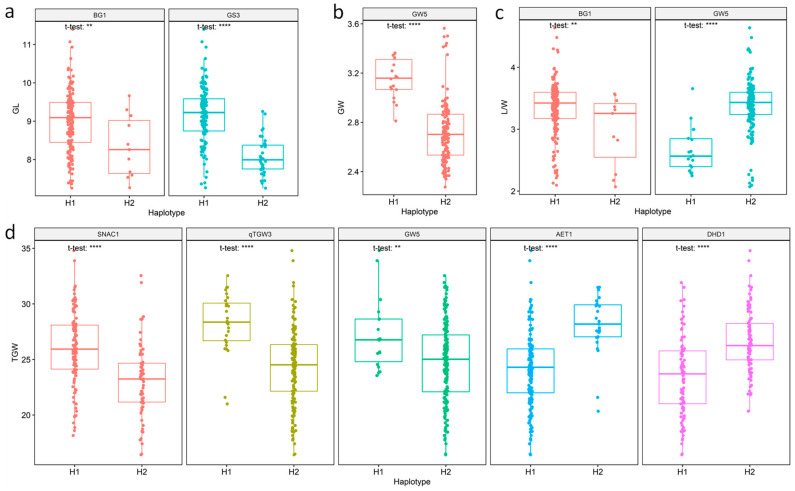
Haplotype analysis of each gene associated with different traits. Boxplots show haplotype diversity and variation between haplotypes of each gene associated with GL (**a**), GW (**b**), L/W (**c**) and TGW (**d**). ** *p* < 0.01, **** *p* < 0.0001.

Furthermore, haplotypes of the seven associated genes were analyzed in the natural population. Except for these haplotypes, which could only be detected in fewer than 1% of these accessions, all seven genes were divided into two haplotypes according to their sequence variation (Table S5). For the two haplotypes of the GL-associated gene *BG1*, *BG1*-H1 could be found in 95% (171) of the accessions, while *BG1*-H2 could only be detected in 5% of these accessions. The average GL showed a significant difference (*p* = 0.0064) between the two haplotypes, which was 9.01 and 8.34 mm, respectively, with *BG1*-H1 being the superior haplotype. Different haplotypes of *BG1* could explain 3.24% of the genetic variation in GL. For *GS3*, another GL-associated gene, the percentages of *GS3*-H1 and *GS3*-H2 were 81.11% and 18.89%, and their average GLs were 9.19 and 8.08 mm, respectively. *GS3*-H1 was the superior haplotype, and different haplotypes of *GS3* could explain 27.66% of the genetic variation in GL (Table 1, Figure 3a).

For the GW-associated gene *GW5*, *GW5*-H1 and *GW5*-H2 accounted for 10% and 86.11% of the accessions investigated, and the average GW of the two haplotypes showed a significant difference (*p* = 2.2 × 10^−12^), measuring 3.15 and 2.72 mm, respectively. *GW5*-H1 was the superior haplotype for GW, and the different haplotypes of *GW5* could explain 25.19% of the genetic variation in GW (Table 1, Figure 3b).

*BG1* and *GW5* mentioned above were also L/W-associated genes. Consistent with the results for GL, *BG1*-H1 was also the superior haplotype for L/W, and this gene could explain 3.21% of the genetic variation in L/W. The average L/W of *BG1*-H1 and *BG1*-H2 also showed significant differences (*p* = 0.0076), measuring 3.35 and 2.98, respectively. However, in contrast to the results for GW, *GW5*-H2 rather than *GW5*-H1 was the superior haplotype for L/W, and the average L/W of accessions with *GW5*-H2 was 3.39, which was significantly larger (*p* = 7.1 × 10^−12^) than that of accessions with *GW5*-H1 (2.66). Different haplotypes of *GW5* could explain 21.1% of the genetic variation in L/W (Table 1, Figure 3c).

Among the five genes associated with TGW, *AET1* was the most dominant gene responsible for TGW, explaining 15.01% of the genetic variation, followed by *SNAC1* (14.78%), *qTGW3* (13.48%), *DHD1* (10.78%) and *GW5* (5.88%). As expected, the average TGWs of the two haplotypes of the five genes were significantly different (*p* values ranging from 2.9E-10 to 0.0055). As shown in Table 1 and Figure 3d, the average TGW of haplotype H1 and haplotype H2 of *AET1*, *SNAC1*, *qTGW3*, *DHD1* and *GW5* was 24.17 g vs. 28.06 g, 28.28 g vs. 24.3 g, 27.26 g vs. 24.78 g, 23.45 g vs. 26.65 g and 27.26 g vs. 24.78 g, respectively. The haplotypes *AET1*-H2, *SNAC1*-H1, *qTGW3*-H1, *DHD1*-H2 and *GW5*-H1 were identified as superior haplotypes for TGW.

### 3.6. Identification of Superior Haplotype Combinations

Since GL, L/W and TGW were associated with two or more genes, the combinations of different haplotypes of different genes and their associations with these traits were then analyzed to illustrate their pyramiding in rice evolution and selection.

**Figure 4:**
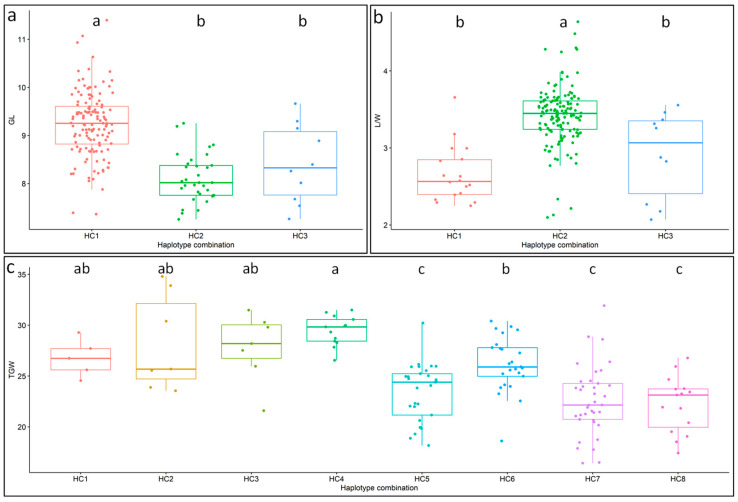
Haplotype combination analysis of genes associated with different traits. Boxplots show haplotype combination diversity and variation between haplotype combinations of the genes associated with GL (**a**), L/W (**b**) and TGW (**c**), and different letters denote significant differences between haplotype combinations.

For the GL-associated genes *BG1* and *GS3*, three haplotype combinations were identified in the 180 accessions, including GL-HC1 (*BG1*-H1, *GS3*-H1), GL-HC2 (*BG1*-H1, *GS3*-H2) and GL-HC3 (*BG1*-H2, *GS3*-H1). The percentages of the three haplotype combinations in these materials were 75.56%, 18.33% and 5.56%, respectively. The average GLs of the three haplotype combinations were also significantly different, measuring 9.24 mm, 8.09 mm and 8.41 mm, respectively. Therefore, GL-HC1 was the superior haplotype combination for GL. The amount of genetic variation explained by both *BG1* and *GS3* was 25.38% (Table 2, Figure 4a).

Three haplotype combinations were also detected for the L/W-associated genes *BG1* and *GW5*. L/W-HC1 (*BG1*-H1, *GW5*-H1), L/W-HC2 (*BG1*-H1, *GW5*-H2) and L/W-HC3 (*BG1*-H2, *GW5*-H2) could be found in 10%, 82.78% and 5.56% of the 180 accessions, respectively. Further analysis showed that the average L/W of L/W-HC2 was significantly different from that of L/W-HC1 and L/W-HC3, but there was no significant difference between the last two haplotype combinations. L/W-HC2 was the superior haplotype combination for L/W, with an average L/W of 3.42, while that of L/W-HC2 and L/W-HC3 was only 2.66 and 2.91, respectively. The two genes together could explain 5.5% of the genetic variation in L/W (Table 2, Figure 4b).

Among the eight haplotype combinations for the five TGW-associated genes, the maximum average TGW (29.47 g) was observed for TGW-HC4, which was a combination of the superior haplotypes for the four TGW-related genes, *SNAC1*-H1, *qTGW3*-H1, *AET1*-H2 and *DHD1*-H2. However, only 6.67% of the 180 accessions had this haplotype combination. Except for TGW-HC4, it was interesting that the other superior haplotype combinations for TGW, including TGW-HC1, TGW-HC2, TGW-HC3 and TGW-HC6, were combinations of superior haplotypes of at least two genes, while the average TGW of the other three haplotype combinations with only one or no superior haplotype of the five genes, including TGW-HC5, TGW-HC7 and TGW-HC8, was significantly lower than the TGW of the five superior haplotype combinations. The eight haplotype combinations explained 22.49% of the total genetic variation in TGW (Table 2, Figure 4c).

### 3.7. Tracing of the Superior Haplotype Combination forThousand-Grain Weight in Past Breeding Practices

**Figure 5:**
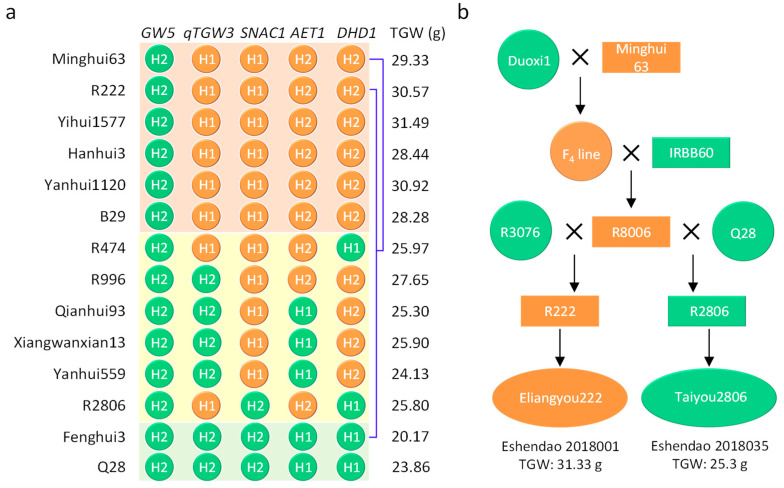
Haplotypes and pedigree of the varieties and lines derived from Minghui63. (**a**) Haplotype and haplotype combinations of the five genes related to TGW in the varieties derived from Minghui63. The orange circle indicates the superior haplotype of the gene, the green circle indicates the non-superior haplotype of the gene, the pink square indicates the varieties containing HC4, the yellow square indicates the varieties containing the superior haplotype of two or three of the five genes, the light green square indicates the varieties not containing the superior haplotype for the five genes, and the blue line indicates the varieties derived from Minghui63. (**b**) Pedigree of the two registered varieties derived from Minghui63. Orange indicates the varieties or lines that contain the superior haplotype combination HC4, while green indicates the varieties or lines that do not contain HC4.

Furthermore, to illustrate the application and validate the effect of the superior haplotypes and haplotype combinations identified in the present study, we analyzed the haplotype combinations of the five TGW-related genes in some other varieties and lines. It was interesting that out of the 12 varieties and lines derived from Minghui63, five contained the same superior haplotype combination HC4 as Minghui63, with an average TGW of 29.94 g. For the other six varieties or lines containing two or three superior haplotypes for the five genes, the average TGW was 25.79 g. However, the TGW of Fenghui3 was only 20.17 g; Fenghui3 does not contain any of the superior haplotypes for the five genes (Figure 5a).

On the other hand, R222 and R2806 are restorer lines that have been used in hybrid rice breeding in the Hubei Province of China, which have been used in cultivating Eliangyou222 (number of registration: Eshendao 2018001) and Taiyou2806 (number of registration: Eshendao 2018035), respectively. Although both restorer lines were derived from Minghui63 (www.ricedata.cn), R222, which had the superior haplotype combination HC4, and its hybrid variety Eliangyou222, showed TGWs of 30.57 g and 31.33 g, respectively. However, the TGWs of R2806, which did not have HC4, and its hybrid variety Taiyou2806 were only 25.80 g and 25.30 g, respectively (Figure 5b).

Moreover, the haplotype and haplotype combinations were also analyzed in our breeding lines and varieties with known pedigrees based on the developed specific markers (Table S6). Three of the four sister lines derived from R3076 and R8006, namely, R171, R222 and R220, had the superior haplotype combination HC4, and their TGWs were 30.76 g, 30.57 g, and 30.24 g, respectively. However, the TGW of the other line (R304) was only 26.86 g; R304 did not have the superior haplotype combination HC4, as expected. In addition, the TGWs of Eliangyou171 and Eliangyou222, hybrid combinations of R171 and R222, were 30.71 g and 31.33 g, respectively (Figure S1).

## 4. Discussion

### 4.1. The Balance of Grain Length and Grain Weight Is Important for Rice Breeding

The grain size of rice is not only crucial for grain weight and yield but also important for grain quality and commercial value. People from different regions tend to choose different types of grain sizes during their daily lives. For example, consumers from America, Southeast Asia and South China like rice with slender grains, while consumers from Japan, Korea and North China prefer rice with short and round grains [2]. Therefore, the improvement of grain shape is always one of the most important factors in rice breeding and the market.

In the present study, 180 *Indica* rice accessions were selected from 3000 germplasms, which included restorer lines, landraces, advanced lines and modern cultivars from all over the world. The GL, GW, L/W and TGW of these 180 *Indica* accessions were investigated in two years. TGW showed higher positive correlations with GW than with GL. GL showed a significant negative correlation with GW. As expected, G/L showed significant positive and negative correlations with GL and GW, respectively. These results suggested that GW played a much more important role in determining TGW than GL. However, grains with a large L/W are popular in the market, such as the varieties selected in South China, most of which are of the slender type, with L/W values greater than 3.5 and TGW values of approximately 22 g (www.ricedata.cn/variety/identified/gdds_1.htm (accessed on 25 Februry 2023)). Therefore, increasing GL at a certain GW level can not only ensure the appropriate TGW but also improve L/W and thus might be important for improving the yield and grain shape for the market.

### 4.2. Candidate Gene-Based Association Analysis Is Useful for Identifying New Functions and Favorable Alleles of Old Genes

In this study, two years of phenotypic data for GL, GW, L/W and TGW and their BLUP values were subjected to candidate gene association analysis, and the variation sites that were identified in both years were considered to be associated with the target traits (*p* < 0.01). Thus, the influence of environmental effects was minimized, and reliable data were generated for further analysis.

Among the 69 associated SNPs/Indels detected, most were located in promoter or exon regions of the seven genes, which may be crucial for the maintenance of their functions or interaction with upstream factors, suggesting that these nucleotide(s) may be functional sites of these genes.

Furthermore, three of the seven genes are known grain size-related genes, including *BG1*, *GW5* and *GS3* [4,11,34]. *GS3* was the first grain-size gene that was cloned by map-based cloning. The C-A substitution in the second exon of *GS3* results in a premature stop codon (TGA); thus, the PEBP domain is truncated, and three other functional domains are missing. Then, a nonfunctional protein is generated [4]. Our analysis also proved that the C-A substitution (*GS3*:1633441) mentioned above was indeed significantly associated with GL. These results also indicated that the associated sites identified in the present study were most likely the functional sites of the target genes, which may be valuable in marker-assisted selection.

Moreover, variations in some other genes were also identified to be associated with the grain character of rice in the current study. For example, *SNAC1* is an abiotic stress-regulated gene. A previous study showed that *SNAC1* mainly contributed to the increased panicle length and number in transgenic plants under drought stress compared with the wild type [35,36]. Our results revealed an SNP in the promoter region of *SNAC1* to be significantly associated with TGW, indicating that *SNAC1* may take part in rice yield potential under normal conditions as well. *AET1* encodes a tRNA^His^Guanylyltransferase and contributes to the modification of pre-tRNA^His^, which is required for normal growth under high-temperature conditions in rice [37]. A SNP in its promoter was found to be associated with TGW. Moreover, overexpression of *DHD1* delayed the heading date of rice but increased the grain length, tiller number, grain number per spike and final yield [38]. An SNP in the promoter of *DHD1* was also identified to be associated with TGW. Although much work needs to be conducted to validate the function of these genes in rice TGW and the associated SNPs in breeding, the results still provide us with a new scope for understanding the yield formation of rice.

### 4.3. Selection during Breeding May Lead to an Imbalanced Distribution of Different Haplotypes and Haplotype Combinations

In this study, only two haplotypes could be detected in the test population for all seven genes (haplotypes that could be detected in fewer than three accessions were excluded). It was probably because most of the accessions in this study were breeding lines, which may have experienced a long period of selection and domestication and resulted in a narrow gene pool used in rice breeding. However, the frequency of the two haplotypes for all the genes was quite imbalanced, with the superior haplotype being the dominant haplotype. For example, in the single-gene haplotype analysis, the frequencies of the superior haplotype and the other haplotype of *GS3* were 81.11% and 18.89%, and the frequencies of the two haplotypes of *GW5* were 88.33% and 10%, respectively.

However, this phenomenon was not observed in the haplotype combination analysis. For instance, the highest frequency was observed for the haplotype combination HC7, which accounted for 20.56% of all eight haplotype combinations in TGW. The frequency of the superior haplotype combination HC4 was only 6.67%. Moreover, the average TGWs of HC7 and HC4 were 22.60 g and 29.47 g, respectively. It may be that grains with large L/W and small TGW values are popular in the market, as mentioned above, and these haplotypes and haplotype combinations have been selected according to the breeding objectives during the breeding process. These results suggest that more attention should be given to these superior haplotype combinations to take full advantage of them in future breeding.

### 4.4. Haplotype Combination 4 forThousand-Grain Weight Can Be Used to Improve Tgw by Mas

TGW-HC4 is the best haplotype combination for TGW, according to our analysis. Our further analysis showed that Minghui63, which is an elite restorer line with the most hybrid combinations in China, has TGW-HC4. It is very striking that a series of varieties or restorer lines derived from Minghui63 also have TGW-HC4, such as Yanhui1120, B29, Hanhui3, Yihui1577 and R222. The average TGW of all these varieties or restorer lines was more than 29.94 g, which is comparable to that of Minghui63 (29.33 g) (Figure 5a). This indicates that TGW-HC4 has been selected and preserved during conventional breeding. Moreover, a number of our own lines and varieties were also genotyped with haplotype combination-specific markers for TGW (Figure 5b and Figure S1). The results also confirmed that TGW-HC4 is important for TGW and can be used in MAS of TGW to improve breeding efficiency. Unfortunately, we did not detect the combination of all five superior haplotypes of TGW-related genes in the current rice panel. We will aim to pyramid the five superior haplotypes together to study their effects on TGW in our future work.

## 5. Conclusions

In the present study, the candidate gene-based association analysis targeting 42-grain size- and weight-related genes in a panel of 180 rice accessions was conducted based on targeted capture sequencing. In total, 69 SNPs/Indels in seven of these 42 genes were detected to be associated with the four grain-related traits investigated. Superior haplotypes and haplotype combinations for the seven genes were also identified. Furthermore, using the haplotype-specific marker panel for the superior haplotype combination TGW-HC4, it was analyzed in Minghui63 and some widely used restorer lines derived from Minghui63, as well as our breeding varieties and lines. The results showed that the TGW-HC4 was indeed the superior haplotype combination for TGW, and it could be utilized in rice breeding. The method will be valuable and practicable for crop molecular design breeding in the future.

## Figures and Tables

**Table 1:** List of grain size- and weight-related genes and haplotypes.

Trait	Gene	Locus ID	Haplotype	Percent of Accessions (%)	Mean	*R*^2^ (%)	*p* Value
GL	*BG1*	LOC_Os03g07920	H1	95.00	9.01	3.24	1.62 × 10^−2^
(mm)			H2	5.00	8.34		
	*GS3*	Os03g0407400	H1	81.11	9.19	27.66	4.04 × 10^−12^
			H2	18.89	8.08		
GW	*GW5*	LOC_Os05g09520	H1	10.00	3.15	25.19	4.90 × 10^−11^
(mm)			H2	88.33	2.72		
L/W	*BG1*	LOC_Os03g07920	H1	95.00	3.35	3.21	1.66 × 10^−2^
			H2	5.00	2.98		
	*GW5*	LOC_Os05g09520	H1	10.00	2.66	21.1	2.58 × 10^−9^
			H2	88.33	3.39		
TGW	*SNAC1*	LOC_Os03g60080	H1	60.56	26.05	14.78	8.36 × 10^−7^
(g)			H2	37.78	23.09		
	*qTGW3*	LOC_Os03g62500	H1	15.56	28.28	13.48	2.63 × 10^−6^
			H2	83.33	24.39		
	*GW5*	LOC_Os05g09520	H1	10.00	27.26	5.88	1.73 × 10^−3^
			H2	88.33	24.78		
	*AET1*	LOC_Os05g45890	H1	78.89	24.17	15.01	6.83 × 10^−7^
			H2	20.00	28.06		
	*DHD1*	LOC_Os11g47920	H1	51.11	23.45	10.78	2.73 × 10^−5^
			H2	46.67	26.65		

**Table 2:** List of grain size- and weight-related haplotype combinations.

Trait	Haplotype Combination	Single-Gene Haplotype	Percentage (%)	Mean	*R*^2^ (%)	*p* Value
GL	HC1	*BG1*-H1, *GS3*-H1	75.56	9.24	25.38	3.96 × 10^−13^
(mm)	HC2	*BG1*-H1, *GS3*-H2	18.33	8.09		
	HC3	*BG1*-H2, *GS3*-H1	5.56	8.41		
L/W	HC1	*BG1*-H1, *GW5*-H1	10	2.66	5.5	9.79 × 10^−4^
	HC2	*BG1*-H1, *GW5*-H2	82.78	3.42		
	HC3	*BG1*-H2, *GW5*-H2	5.56	2.91		
TGW (g)	HC1	*SNAC1*-H1, *qTGW3*-H2, *GW5*-H1, *AET1*-H1, *DHD1*-H1	2.78	26.78	22.49	2.53 × 10^−9^
	HC2	*SNAC1*-H1, *qTGW3*-H2, *GW5*-H1, *AET1*-H1, *DHD1*-H2	3.89	28.25		
	HC3	*SNAC1*-H1, *qTGW3*-H1, *GW5*-H2, *AET1*-H2, *DHD1*-H1	3.33	27.84		
	HC4	*SNAC1*-H1, *qTGW3*-H1, *GW5*-H2, *AET1*-H2, *DHD1*-H2	6.67	29.47		
	HC5	*SNAC1*-H1, *qTGW3*-H2, *GW5*-H2, *AET1*-H1, *DHD1*-H1	16.11	23.39		
	HC6	*SNAC1*-H1, *qTGW3*-H2, *GW5*-H2, *AET1*-H1, *DHD1*-H2	13.89	26.19		
	HC7	*SNAC1*-H2, *qTGW3*-H2, *GW5*-H2, *AET1*-H1, *DHD1*-H1	20.56	22.6		
	HC8	SNAC1-H2, *qTGW3*-H2, *GW5*-H2, *AET1*-H1, *DHD1*-H2	7.78	22.22		

## Data Availability

The datasets generated during or analyzed during the current study are available from the corresponding author upon reasonable request.

## Supplementary Materials

The following supporting information can be downloaded at https://www.mdpi.com/article/10.3390/genes14122201/s1. Figure S1: pedigree and haplotype combinations related to TGW of the lines and varieties that we have developed; Table S1: list of material information; Table S2: list of sequenced genes and related information; Table S3: average variation and heritability of grain-related traits in rice across different environments; Table S4: list of trait-associated genes identified by candidate gene-based association analysis; Table S5: sequence variations of the genes associated with the traits; Table S6: information on haplotype-specific molecular marker primers for the genes associated with TGW.
