# Supplementary material for: Identification of Superior Haplotypes and Haplotype Combinations for Grain Size- and Weight-Related Genes for Breeding Applications in Rice (Oryza sativa L.)
Source: Genes (Basel). 2023 Dec 12;14(12):2201. doi: 10.3390/genes14122201 (PMC10742856; doi:10.3390/genes14122201)
Supplement: Supplementary file 1 [file genes-14-02201-s001.zip › Figure S1.pdf]

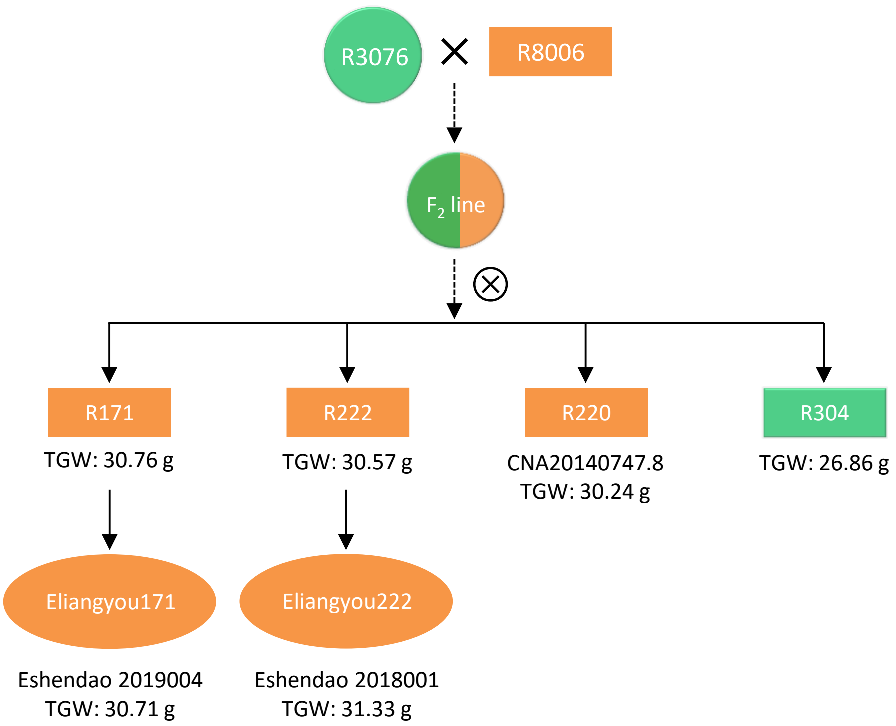

Figure S1. Pedigree and haplotype combinations related to TGW of the lines and varieties that we have developed. Yellow indicated that the varieties or lines which contain the superior haplotype-combination HC4, green indicated that the varieties or lines which do not contain the superior haplotype-combination HC4.
